# Reply to Vural, Ü. Comment on “Akkaya et al. Posterior Teflon-Felt-Reinforced Coronary Button Anastomosis in a Modified Bentall Procedure: Early Outcomes in a Single-Center Retrospective Study. *J. Clin. Med.* 2026, *15*, 2546”

**DOI:** 10.3390/jcm15218622

**Published:** 2026-11-05

**Authors:** Özgür Akkaya, Izatullah Jalalzai, Ümit Arslan

**Affiliations:** 1 Department of Cardiovascular Surgery, Faculty of Medicine, Alanya Alaaddin Keykubat University, Antalya 07450, Türkiye; 2 Department of Cardiovascular Surgery, Faculty of Medicine, Atatürk University, Erzurum 25030, Türkiye; ijalalzai@gmail.com

We thank Dr. Vural for his careful reading of our article [1] and constructive comments [2]. Before addressing the individual points, we would like to reiterate the interpretation and limitations already stated in our original report. Our retrospective, single-center study included a relatively small, selected cohort of patients undergoing elective modified Bentall procedures. We acknowledged that the small sample size may limit statistical power, that the single-surgeon setting limits generalizability, and that our assessment was confined to early postoperative outcomes. We also noted that long-term coronary patency and anastomotic durability remain unknown. Accordingly, our findings were presented as preliminary observations suggesting the potential technical value of posterior Teflon-felt reinforcement, with confirmation in larger studies required. Dr. Vural’s comments provide an opportunity to clarify these limitations and the interpretation of specific outcomes.

Our original study reported fewer re-explorations in the reinforced group, but the between-group difference was not statistically significant. Importantly, overall re-exploration is not synonymous with coronary button bleeding. Of the nine re-explorations in the conventional group, two were related to coronary button bleeding; no coronary button-related bleeding was observed among the three re-explorations in the reinforced group. These observations cannot establish that reinforcement prevents bleeding, and other potential sources of postoperative hemorrhage must be considered.

As acknowledged in the original article, the single-center, single-surgeon, retrospective design and limited sample size restrict the strength and generalizability of the findings. In addition, the conventional and reinforced techniques were used during different time periods because the technique evolved over the course of the study. Changes in surgical experience and perioperative practice could therefore have influenced the observed outcomes. Data were obtained from the institutional cardiovascular surgery database and supplemented by the operating surgeon’s prospectively maintained logbook; this does not eliminate the limitations inherent to a retrospective, noncontemporaneous comparison.

The original article described lower blood-product use in the reinforced group, alongside a trend toward less 24 h chest tube drainage. These are early postoperative associations and should not be attributed to the coronary button technique alone. Transfusion requirements can also reflect perioperative management and other factors not isolated by our study design. Our results therefore support further investigation rather than a conclusion of a causal reduction in blood loss or transfusion.

Our original report focused on early clinical outcomes and explicitly identified the absence of long-term coronary patency and anastomotic durability data as a limitation. Routine postoperative coronary CT angiography or invasive coronary angiography was not performed; coronary button integrity was assessed intraoperatively and through postoperative clinical surveillance. Consequently, the data do not demonstrate anatomical superiority or durable coronary patency. Future assessment should incorporate objective coronary imaging and longer follow-up.

The technical rationale for posterior felt reinforcement includes providing support to the coronary button during anastomosis. However, the original study excluded acute aortic dissection to reduce clinical heterogeneity, and tissue fragility was not independently assessed. A specific benefit in fragile or dissected tissue therefore remains hypothetical and cannot be inferred from the present cohort.

Conclusions: Consistent with the limitations already discussed in our published article, we regard these findings as preliminary early observations of the feasibility and potential usefulness of posterior Teflon-felt-reinforced coronary button anastomosis. They do not establish a causal reduction in bleeding, superior coronary outcomes, or an indication for routine adoption. Larger, preferably contemporaneous comparative studies with standardized perioperative management, objective coronary imaging, and longer follow-up are necessary to determine the clinical value of this modification.

We thank Dr. Vural for the opportunity to clarify these aspects of our report.

## Data Availability

The datasets underlying the original article are available from the corresponding author upon reasonable request.
